# TMPRSS2 activity may mediate sex differences in COVID-19 severity

**DOI:** 10.1038/s41392-021-00513-7

**Published:** 2021-03-01

**Authors:** Derick Okwan-Duodu, Eun-Cheon Lim, Sungyong You, David M. Engman

**Affiliations:** 1 Departments of Biomedical Sciences, Cedars Sinai Medical Center, Los Angeles, CA USA; 2 Department of Pathology & Laboratory Medicine, Cedars Sinai Medical Center, Los Angeles, CA USA; 3 Department of Pathology, Northwestern University, Chicago, IL USA

**Keywords:** Infection, Computational biology and bioinformatics

**Dear Editor**,

Since the initial reports of severe acute respiratory syndrome coronavirus 2 (SARS-CoV-2) infection early this year, coronavirus disease 2019 (COVID-19) has rapidly reached pandemic levels with high transmissibility and low but significant mortality. While individuals with a number of underlying comorbidities are more likely to develop severe disease, male sex has emerged as an independent risk factor for poor prognosis, an observation that cannot be accounted for by age or social behaviors including smoking.^1^ Further, animal models of coronavirus infections also demonstrate differences in disease severity between males and females,^2^ suggesting a bona fide lifestyle-independent sexual dimorphism in disease expression. The mechanistic basis for this remains elusive.

Infection of host cells by SARS-CoV-2 is mediated by direct binding of the viral spike protein (S) to the host surface carboxypeptidase ACE2. Once bound, the host surface serine protease TMPRSS2 promotes viral entry by cleaving the S protein into the S1 (receptor binding) and S2 (membrane fusion) domains, the latter of which is the actual mediator of virus-host membrane fusion.^3^ In an unbiased approach to assess whether ACE2 and TMPRSS2 mediate the observed sex disparity, we analyzed single-cell RNA sequences (scRNA-seq) from the lungs of 13 healthy men (mean age 51.8 ± 4 years) and 13 healthy women (mean age 46.9 ± 6 years) available from three public datasets (GSE122960, GSE130148, and GSE133747). Single-cell expression analysis permits the attribution of mRNA levels to individual cells (and cell types) in the organ. Other scRNA-seq studies involving patients with a significant smoking history or pulmonary disease (pulmonary fibrosis, primary or metastatic lung cancer, severe chronic obstructive pulmonary disease, and others) were excluded from this study even if sequencing was performed on clinically uninvolved lungs, as these complex pathophysiological processes may potentially alter lung homeostasis globally. Data were processed, integrated, and batch-corrected for downstream analysis. Our methodology for preprocessing and integration of scRNA-seq datasets, cell-type assignment, and differential expression analysis are detailed in Supplementary Methods.

Uniform manifold approximation and projection (UMAP) analysis of scRNA-seq datasets from the lungs of 13 males and 13 females was used to assign each cell to 1 of 12 cell types (Fig. 1a, top panel) and none-sex aggregated analysis of *Ace2* and *Tmprss2* revealed the highest mRNA expression in alveolar type (AT) 1 and 2 epithelial cells, with the *Tmprss2* levels being highest (Fig. 1b). The relative mRNA expression levels of *Ace2* did not differ based on sex, but *Tmprss2* expression was significantly increased in males (*P* = 1.592 × 10^−18^, Fig. 1c). More importantly, ACE2^+^ TMPRSS2^+^ double-positive AT2 cells were increased more than threefold in males (odds ratio *P* = 2.143 × 10^−5^, Fig. 1d). These cells are particularly relevant for COVID-19 infection, as evidenced by their marked enrichment for genes mediating viral replication and translation pathways when compared with other AT2 cells (Fig. 1e). Thus, this increased frequency of ACE2^+^ TMPRSS2^+^ AT2 cells may provide the cellular basis for increased COVID disease severity in adult males upon potential exposure to similar viral loads.

**Fig. 1:**
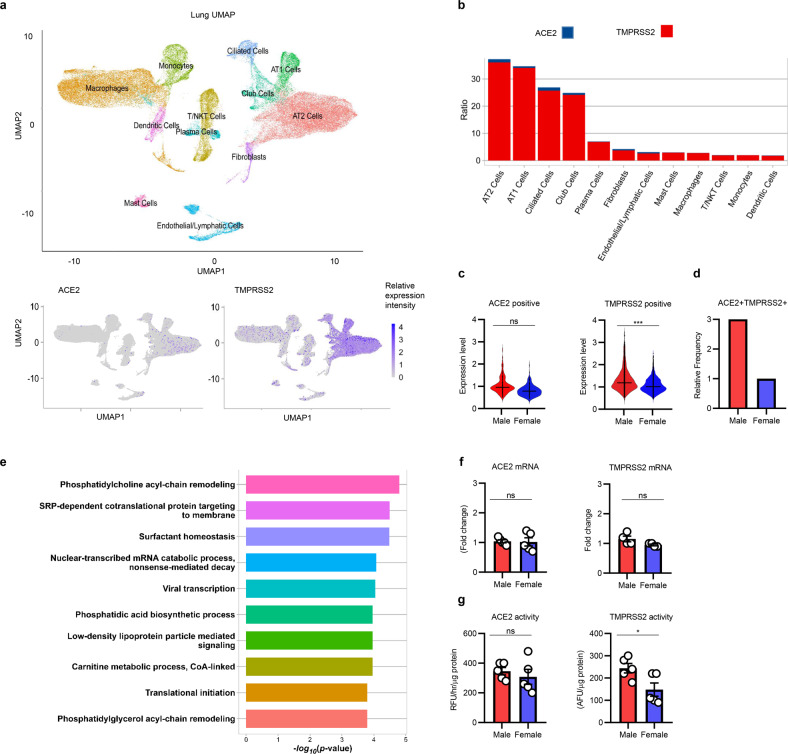
**a** UMAP plot of different cell types in the lungs obtained from scRNA-seq integrated analysis of 13 female and 13 male healthy patients (left), followed by intensity UMAPs of ACE2-expressing cells (bottom left) and TMPRSS2-expressing cells (bottom right). **b** Per cell ratios of *Ace2* and *Tmprss2* gene expression levels across cell types. Ratios were determined by dividing the mRNA expression by the number of cells in each stratified cell type. **c** Violin plots of *Ace2* and *Tmprss2* mRNA expression levels in male and female AT2 cells. **d**, Relative frequency of TMPRSS2^+^ ACE2^+^ double-positive cells in AT2 cells in males and females, normalized to females. *P* <2 × 10^−5^ by chi-square test. **e** Gene set enrichment analysis for TMPRSS2^+^ ACE2^+^ double-positive AT2 cells. Bar plots representing enrichment score of gene sets. **f** *Ace2* and *Tmprss2* mRNA levels in male and female mouse lungs, normalized to females (*n* = 5 per group). **g** Relative ACE2 and TMPRSS2 enzymatic activity levels in male and female mouse lungs (*n* = 5 per group). ns not significant, *P < 0.05, **P < 0.001 by unpaired *t* test (**f**, **g**) and the Wilcoxon rank-sum test (**c**)

We investigated whether the male skewing of the frequency of pulmonary alveolar epithelial cells expressing TMPRSS2 is also true in mice. Six-month-old mice were evaluated, as their age more accurately correlates with that of mature human adults. Experiments were performed in agreement with guidelines approved by the Cedars-Sinai Institutional Animal Care and Use Committee (IACUC). By RT-PCR, mRNA expression of *Ace2* and *Tmprss2* did not differ between male and female mice lungs (Fig. 1f). However, while the enzymatic activity of ACE2 was similar in males and females, TMPRSS2 activity was significantly increased in the lungs of male mice (Fig. 1g).

Our results are consistent with there being little sex dimorphism in ACE2 expression beyond the kidney, despite its known location on the X chromosome. Other resources such as the genotype-tissue expression (GTEx) database analysis have not found consistent differences in ACE2 expression in a sex-dependent manner. The pulmonary behavior of TMPRSS2, an androgen-regulated protease that mediates oncogenic fusion to drive prostate cancer pathogenesis, is more obscure. Interestingly, TMPRSS2 was mainly expressed by AT1 and AT2 epithelial cells and ciliated cells, similar to the pattern for ACE2 but with a larger cellular footprint. However, overall, TMPRSS2 expression was significantly higher in males than in females by scRNA-seq analysis. The fraction of ACE2^+^ TMPRSS2^+^ AT2 cells was approximately threefold higher in males. In mice, although pulmonary TMPRSS2 mRNA levels are similar in males and females, TMPRSS2 enzymatic activity was significantly higher in males. One potential reason for the absence of TMPRSS2 expression level differences in mice is testing methodology, as RT-PCR analyzes the global expression of whole lung tissue. However, even in that setting, the activity of TMPRSS2 was greater in male mice. These results suggest tissue-specific regulation of sex hormone-sensitive proteases. Moreover, androgen regulation of TMPRSS2 may possibly affect not only protein expression, but also protease activity. Further work is warranted to illuminate the link between these host receptors that mediate disease pathogenesis and the immune response against COVID-19 in males and females. Indeed, the role of TMPRSS2 expression and activity in lung immunity remain poorly understood, particularly in the context of emerging efforts to utilize sex steroid-based therapies to improve COVID-19 outcomes. We note that in prostate cancer, the expression of TMPRSS2 correlates with immune infiltration, which affects prognosis.

Epidemiologic studies reveal that other coronavirus infections show greater severity in males. Although the Middle East respiratory syndrome coronavirus (MERS-CoV) entry into the cell is mediated by dipeptidyl peptidase 4 rather than ACE2, all CoV infectivity is uniformly augmented by TMPRSS2 proteolysis. Further, TMPRSS2 is also the major activating protease for Influenza A and B,^4^ in which male sex likewise portends poor outcome.^5^ Our findings provide an important insight into the sexual dimorphism in COVID-19 outcomes and illuminate features of disease pathogenesis relevant for potential therapeutic targeting.

## Supplementary information

Supplementary Data

## Data Availability

The authors confirm that the data supporting the findings of this study are available within the article [and/or] its supplementary materials. Additional details are available upon request.
