# Supplementary material for: TMPRSS2 activity may mediate sex differences in COVID-19 severity
Source: Signal Transduct Target Ther. 2021 Mar 1;6:100. doi: 10.1038/s41392-021-00513-7 (PMC7919249; doi:10.1038/s41392-021-00513-7)
Supplement: Supplementary file 1 — Supplementary Data [file 41392_2021_513_MOESM1_ESM.pdf]

Supplementary Materials for

TMPRSS2 Activity may Mediate Sex Differences in COVID-19 Severity

Derick Okwan-Duodu[1,2#], Eun-Cheon Lim[1], Sungyong You[1], David M. Engman[1,2,3]

Correspondence to: derick.okwan@cshs.org

This PDF file includes:

Materials and Methods

Materials and Methods

Methods

Preprocessing and integration of single cell RNA-seq data sets

We collected twenty-six human single-cell RNA-seq data of thirteen males and thirteen females from three datasets (GSE122960, GSE130148, GSE133747) via Gene Expression Omnibus in National Center for Biotechnology Information (https://www.ncbi.nlm.nih.gov/geo/) [1]. scRNA-seq data from the datasets were pre-processed following the standard workflow of Seurat V3. Cells which have unique feature counts over 10,000 or less than 100 and mitochondrial counts over 15% were filtered to improve the quality of downstream analysis. A normalized expression matrix was created by the LogNormalize method with default settings (a scale factor of 10,000), followed by log-transformation. Sex information and identified variable features or genes were included with individual metadata. The integrated batch corrected expression matrix was scaled according to variable features. The gender difference was assessed with lung tissue RNA-seq

expression data from GTEx portal with following links (ACE2: https://gtexportal.org/home/gene/ACE2; TMPRSS2: https://gtexportal.org/home/gene/TMPRSS2).

Cell-type assignment and differential expression analysis

We performed a principal component analysis (PCA) on the integrated data. The dimensionality of the data was reduced by Uniform Manifold Approximation and Projection (UMAP) with PCA embeddings (k=21). The optimal value of k, which is the number of PCA embeddings, was determined by the Elbow method and fifteen clusters were found. Differentially expressed features were identified with a minimum percentage of 25% in either of the two clusters, and a log-fold change > 0.25. Top-ten features were matched against a marker association catalog between average gene expression differences and cell type identity. We assign each cell type identity to a cluster that maximizes the number of top-ten highly expressed genes. In turn, twelve cell types were annotated from which downstream analysis is performed.

The log fold-change of the average TMPRSS2, and ACE2 expression between male and female group is calculated by the FindMarkers module in Seurat V3. A positive average log-fold change value indicates that the TMPRSS2 gene is more highly expressed in the male group. p-values were computed by the Wilcoxon rank sum test. p-values are adjusted by the Bonferroni correction given an expression matrix of TMPRSS2 and ACE2 features.

Animals

Six-month-old male and female B6129SF1/J littermates (Jackson Laboratory stock # 101043) were used for our analysis. The animals were housed and cared for in agreement with guidelines approved by the Cedars-Sinai Medical Center Institutional Animal Care and Use Committee.

ACE2 activity

ACE2 activity was measured by a fluorogenic assay. In brief, lung tissues were homogenized and after protein quantification by the Pierce BCA assay (ThermoFisher), 10 µg of protein was loaded onto 96-well plate with intramolecularly quenched ACE2-specific substrate Mca-APK-Dnp (Anaspec) according to the manufacturer's recommendation. Fluorescence was measured at 320/405 nm excitation/emission wavelength (FLUOstar Omega). Values were subtracted from ACE2 activity inhibited by 1µM DX600.

TMPRSS2 activity

Briefly, lung tissues were homogenized, and 10 µg of protein was loaded in a reaction buffer (5 mM Tris-HCl, pH 7.75). Samples were incubated for 1 h at 37 ºC with 1 µl of the fluorogenic TMPRSS2-specific substrate Boc-Gly-Gly-Arg-Mca (Fisher Scientific) at a 10 mM concentration. Fluorescence was measured at 360/460 nm excitation/emission wavelength.

RT-PCR

RT-PCR was used to determine lung ACE2 and TMPRSS2 mRNA expression (ddCt relative quantitation). RNA was isolated from lung using TriZol reagent (ThermoFisher). After reverse transcribing templates to obtain cDNA, quantitative PCR was performed using Quantstudio 5

(ThermoFisher). Expression was determined relative to GAPDH. The following primers (Integrated DNA Technologies) were used;

TMPRSS2 5’- CAGTCTGAGCACATCTGTCCT, 3’-CTCGGAGCATACTGAGGCA

ACE2 5’ – GGAGCCTGTCAGGGCTACT, 3’- CCACAAGAATCTGTACCTTCTGC

GAPDH 5’- TGTGTCCGTCGTGGATCTGA, 3’- CCTGCTTCACCACCTTCTTGA

Statistics

Statistical analyses were performed using R (v3.6.3) with Seurat (v3.1.5) and MAST (v1.12.0) packages. For comparisons between cell-specific TMPRSS2 and ACE2 values between males and females, the Wilcoxon rank sum test was used. Gene set enrichment analysis were performed using TopGO (v2.38.1) package. *P* values were adjusted for multiple testing using the standard Bonferroni method. For mouse studies, data are presented as mean ± SEM. Differences between groups were assessed for statistical significance using an unpaired Student’s t test (two-tailed). Analysis were performed using GraphPad Prism 6.04 (GraphPad Software, San Diego, CA). A value of $P \leq 0.05$ was considered significant.

REFERENCE

1. Edgar R, Domrachev M, Lash AE. Gene Expression Omnibus: NCBI gene expression and hybridization array data repository. *Nucleic Acids Res.* 2002;30(1):207-210.
